# Biological and biochemical characterization of HIV-1 Gag/dgp41 virus-like particles expressed in *Nicotiana benthamiana*

**DOI:** 10.1111/pbi.12058

**Published:** 2013-03-19

**Authors:** Sarah A Kessans, Mark D Linhart, Nobuyuki Matoba, Tsafrir Mor

**Affiliations:** 1 School of Life Sciences and The Biodesign Institute, Arizona State UniversityTempe, AZ, USA; 2 Owensboro Cancer Research ProgramOwensboro, KY, USA; 3 James Graham Brown Cancer Center and Department of Pharmacology & Toxicology, University of Louisville School of MedicineLouisville, KY, USA

**Keywords:** HIV-1, enveloped virus-like particles, transgenic plants, transient expression, Gag, gp41

## Abstract

The transmembrane HIV-1 envelope protein gp41 has been shown to play critical roles in the viral mucosal transmission and infection of CD4+ cells. Gag is a structural protein configuring the enveloped viral particles and has been suggested to constitute a target of the cellular immunity that may control viral load. We hypothesized that HIV enveloped virus-like particles (VLPs) consisting of Gag and a deconstructed form of gp41 comprising the membrane proximal external, transmembrane and cytoplasmic domains (dgp41) could be expressed in plants. To this end, plant-optimized HIV-1 genes were constructed and expressed in *Nicotiana benthamiana* by stable transformation, or transiently using a Tobamovirus-based expression system or a combination of both. Our results of biophysical, biochemical and electron microscopy characterization demonstrates that plant cells could support not only the formation of enveloped HIV-1 Gag VLPs, but also the accumulation of VLPs that incorporated dgp41. These findings provide further impetus for the journey towards a broadly efficacious and inexpensive subunit vaccine against HIV-1.

## Introduction

The highly conserved membrane proximal external region (MPER) of the HIV-1 envelope protein gp41 plays important roles during mucosal transmission and target (CD4^+^) cell infection (Ashkenazi and Shai, 2011; Checkley *et al*., 2011; Denner, 2011; Nieva *et al*., 2011). Furthermore, antibodies (Abs) against this region have shown to exhibit broad anti-HIV-1 responses such as viral neutralization and transcytosis blockade (Cai *et al*., 2011; Denner, 2011; Garg *et al*., 2011). Therefore, the MPER is considered to be a potentially important component in a subunit vaccine against the virus. However, this region was shown to be very poorly immunogenic on its own, and the precise presentation of immunologically relevant structure seems to critically affect its vaccine efficacy (Denner, 2011; Matoba *et al*., 2008, 2009, 2011; Nieva *et al*., 2011). In particular, it is likely that the MPER would need to mimic its conformation on the surface of the native HIV-1 virion, and consequently, it needs to be presented in the context of a membrane (Atilgan *et al*., 2001; Cerasoli *et al*., 2012; Postler *et al*., 2012; Visciano *et al*., 2011).

Enclosed membrane vesicles with viral membrane proteins construe one kind of virus-like particle (VLP) and have been demonstrated in the past to be of value as immunogens (e.g. hepatitis B surface antigen (Roldao *et al*., 2010) and Influenza hemagglutinin-based VLPs (D’Aoust *et al*., 2010)). However, HIV-1’s gp41 cannot form VLPs on its own and requires the vesicle-forming function of the main structural protein of the virus, p55^Gag^ (Gag, group-associated antigen of HIV-1. For recent reviews, see Balasubramaniam and Freed, 2011; Briggs and Krausslich, 2011). Therefore, it was hypothesized that enveloped VLPs consisting of Gag that incorporated a deconstructed form of gp41 comprising the MPER, transmembrane domain, and cytoplasmic tail (dgp41) into the membrane of the VLPs may present the MPER in its natural state (Klasse *et al*., 2012).

HIV-1 VLPs have been shown to assemble in mammalian (Hammonds *et al*., 2007; Krausslich *et al*., 1993; Wagner *et al*., 1994), insect (Deml *et al*., 1997, 2004; Jaffray *et al*., 2004; Tagliamonte *et al*., 2010; Visciano *et al*., 2011; Wang *et al*., 2007; Yamshchikov *et al*., 1995) and yeast cells (Morikawa *et al*., 2007; Sakuragi *et al*., 2002). However, expression in plants has been less successful, although plants can successfully express enveloped VLPs from other viruses (e.g. D’Aoust *et al*., 2010). Two recent studies (Meyers *et al*., 2008; Scotti *et al*., 2009) were successful in expressing full-length Gag within the chloroplasts of *Nicotiana benthamiana* and *N. tabacum* cells, but both had difficulty in expressing Gag within the cytoplasm, the compartment in which the biogenesis of VLP begins in animal cells (Meyers *et al*., 2008; Scotti *et al*., 2009).

In this study, we demonstrate *in planta* assembly and budding of HIV-1 VLPs consisting of full-length p55^Gag^ and a deconstructed variant of gp41 (dgp41)—comprised of its MPER, transmembrane domain and cytoplasmic tail. Co-expression of plant-optimized recombinant genes encoding these proteins was achieved through the novel combination of traditional stable nuclear transformation and a tobamovirus-based transient over-expression system.

## Results

### *De novo* construction of plant-expression-optimized gag and dgp41 genes

**Figure 1:**
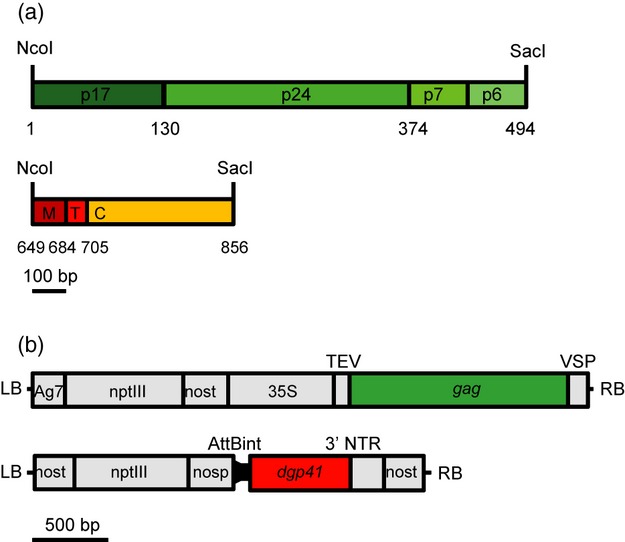
Expression cassettes for synthetic genes encoding p55^Gag^ and dgp41. (a) Gag (top) and dgp41 (bottom) constructs, showing domain regions of the corresponding protein, as well as codon number of native HIV-1 gene. (b) T-DNA construct of *gag* in pGPTV-Kan (LB – left T-DNA border, Ag7 – Agrobacterium gene 7 poly-A signal, nptIII – kanamycin resistance gene, nos t – nos terminator, 35S – cauliflower mosaic virus 35S promoter, TEV – tobacco etch virus 5′ untranslated region, VSP – polyadenylation signal of soybean vegetative storage protein gene, RB – right T-DNA border) and T-DNA construct of *dgp41* in the 5′ module of the MagnICON system (AttB – recombination site, int – intron, NTR – nontranslated region). Scale for (a and b) at the bottom of the figure.

To increase the level of transcription of the two HIV-1 genes under the two expression modalities explored here, stable transgenic expression was driven by the strong constitutive cauliflower mosaic virus 35S promoter (Figure 1) and by the vigorous activity of turnip vein-clearing virus’s RNA-dependent RNA polymerase (RdRp) of the MagnICON transient expression system (Marillonnet *et al.*, 2004, 2005). In addition, to reduce the extent of DNA methylation of the *gag* transgene, associated in plants with transcriptional gene silencing, 49 of the 85 potential methylation sites present in the native gene were abolished by silent mutations introduced into the plant-optimized version of the gene (Table S1).

Beyond transcription rates, the accumulation of recombinant proteins in plants can be greatly affected by post-transcriptional, translational and post-translational events. To this end, we have removed from the plant-expression-optimized gag gene all 30 spurious splicing signals (Hebsgaard *et al.*, 1996), cryptic polyadenylation sites (Loke *et al.*, 2005) and mRNA destabilizing sequences with which the native sequence was ridden (Figure S1, Table S1). Similarly, 14 such deleterious sequences were removed from the sequence of the plant-optimized dgp41 sequence (Figure S2, Table S1).

Translatability of foreign gene transcripts can be increased by replacing unfavourable codons with synonymous codons that are more widely used in plant genes of highly expressed proteins. Our analyses demonstrated that approximately a third of the codons in the native *gag* and *dgp41* genes were unfavourable for expression in plants (33% and 32% of the codons, respectively, had *w*< 0.5, for definition see the Experimental Procedures section below). In the plant-expression-optimized versions of the *gag* and *dgp41* genes, the majority of the unfavourable codons have been eliminated reducing their occurrence, respectively, to 7% and 2% (Figure S1, S2, Table S1). In accordance with these results, the calculated codon adaptiveness (CAI) increased from 0.5 to 0.8 (*gag*) and from 0.6 to 0.9 (dgp41) similar to the CAI value of the small subunit of ribulose-1,5-bisphosphate carboxylase oxygenase (RuBisCO, CAI value = 0.8), a nuclear encoded protein that accumulates to very high levels (Figure S3).

### Gag transient and stable expression

**Figure 2:**
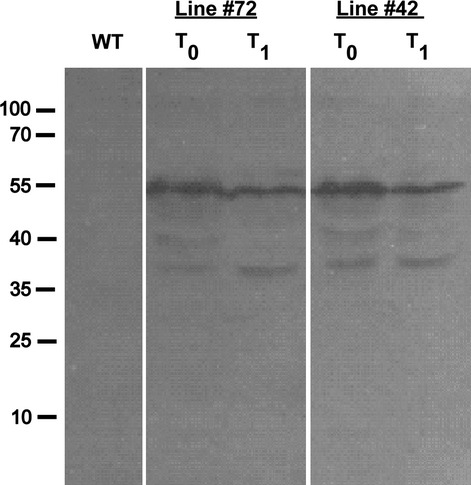
Expression of Gag in transgenic plants. WT – Wild-type plant extract. Two lines (#72 and 42) were created. *T*_0_ is the first generation, *T*_1_ is the second generation for both of those lines. Immunoblot developed with α-p24 antibodies.

Following *Agrobacterium*-mediated transformation of more than 300 explants, 100 of which regenerated into kanamycin resistant plants, we could identify only two independent transformants that expressed the Gag protein. Remarkably, these two plant lines were phenotypically indistinguishable from wild-type plants, at least under normal greenhouse growth conditions. Crude water-soluble protein extracts were resolved by SDS-PAGE followed by immunoblotting (Figure 2), and the analysis indicated the presence of a major band with apparent molecular mass of ∼55 kD corresponding to the full-length p55^Gag^ protein. In addition, crude extracts almost always contained discrete lower molecular bands corresponding to either cleavage products of the polyprotein or to partial-translation products. Using quantitative immunoblot analysis with Abs raised against the p24 moiety of the polyprotein (Figure 1), we determined that the stably expressed full-length Gag accumulated to ∼22 mg/kg fresh weight.

### Transient expression of dgp41

**Figure 3:**
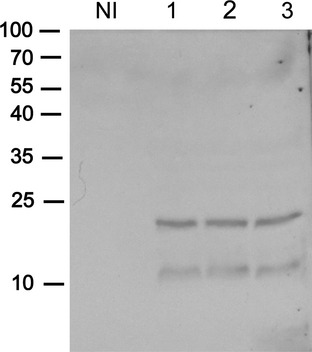
Transient expression of dgp41. NI – Noninfiltrated wild type. 1, 2 and 3 are three samples of three independently infiltrated plants that were harvested 5 days postinfiltration. Immunoblot developed with 2F5 antibodies.

The deconstructed gp41 was first expressed in wild-type *N. benthamiana* plants using an ICON apoplastic targeting module containing the barley alpha-amylase signal peptide (pICH20999, Kalthoff *et al.*, 2010). Expression was confirmed using immunoblotting (Figure 3), showing a prominent band at 24 kD, the expected size of the dgp41 protein, as well as a smaller degradation product containing the MPER fragment (∼10 kD). As expected, targeting the recombinant protein to the cytoplasm did not allow its accumulation to detectable levels (data not shown). Interestingly, using alternative 5′-modules equipped with different signal peptides, including those of apple pectinase, rice alpha-amylase and *N. plumbagenifolia* calreticulin, resulted in drastically lower accumulation levels that were below the sensitivity of our immunoblot assay.

**Figure 4:**
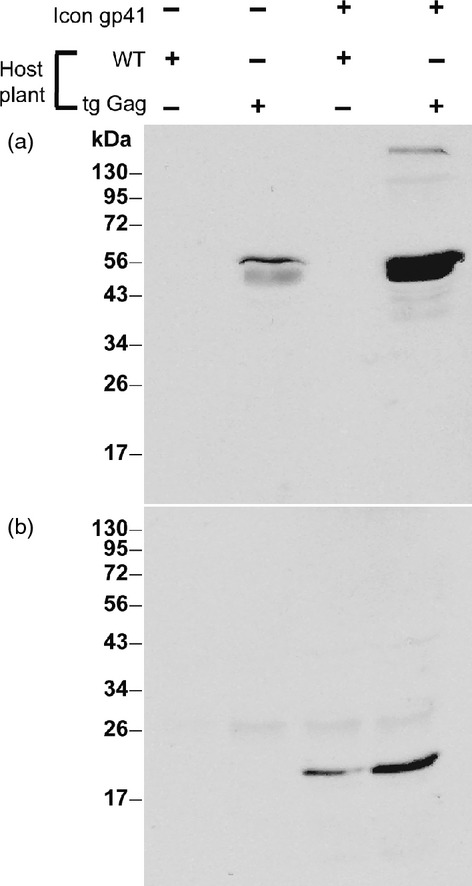
Co-expression of Gag and dgp41. The co-expression of Gag and dgp41 seemed to stabilize the expression of both proteins as compared to the expression of either protein by itself, suggesting that the two proteins might be interacting with each other, possibly in the context of a VLP.

Once expression was confirmed in wild-type plants, stable lines expressing the Gag protein were infiltrated with the dgp41 MagnICON constructs. Co-expression of the proteins in these plants was confirmed by immunoblotting (Figure 4). Interestingly, both proteins accumulated to higher levels when co-expressed as compared to their expression on their own, suggesting a mutual costabilization effect that allowed their accumulation to higher levels—2.3-fold and 2.4-fold increase for dgp41 and Gag, respectively. Our results are congruent with previous reports about the costabilization of multimeric proteins in plants (e.g. monoclonal antibodies, Hiatt *et al.*, 1989). Quantification of gp41 when co-expressed with stably expressed Gag was determined by quantitative immunoblot to be ∼9 mg/kg fresh leaf weight.

### Cosedimentation of Gag and dgp41

**Figure 5:**
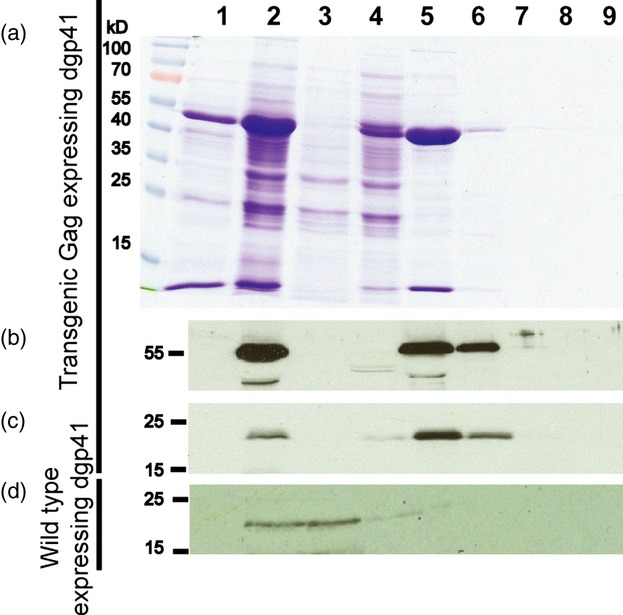
Cosedimentation of Gag and dgp41. Plant water-soluble extracts were subjected to 10%–60% iodixanol gradient centrifugation. Individual fractions were collected, subjected to SDS-PAGE (a) followed by blotting and immunodecoration with anti-Gag Abs (b) or anti-MPR Abs (c). In a separate experiment, extracts were obtained from WT plants transiently expressing dgp41 (in the absence of Gag) and were similarly treated as described above (d). Lane 1: Wild-type control. Lane 2: Whole extract Lane 3: water-soluble clarified extract. Lane 4–9: 10%, 20%, 30%, 40%, 50% and 60% iodixanol fractions.

The costabilization of Gag dgp41 (or their more efficient extraction) observed when the proteins are co-expressed, suggests that they may be interacting with each other, as indeed happens within the HIV-1 viral particle (Brandano and Stevenson, 2011; Weclewicz *et al.*, 1998). Virions and VLPs have characteristic sizes and densities and can be separated from other cellular components by rate-zonal ultracentrifugation employing preformed density gradients. We subjected clarified homogenates of plant extracts to optiprep® (iodixanol) step-gradient centrifugation (10%–60%), and results demonstrated that most, but not all of the Gag and dgp41 proteins migrated well into the gradients and could be recovered in the 20%–30% fractions (Figure 5). Similar results were previously reported for HIV-1 VLPs from other sources such as insect cells (Buonaguro *et al.*, 2001) and yeast (Sakuragi *et al.*, 2002). It is important to note that when expressed by itself, dgp41 has a different fractionation pattern (Figure 5d). The protein can be found mostly in the 0%–20% fractions suggesting a different mode of association of the protein in the absence of its Gag partner.

### Plant-produced HIV-1 VLPs are enveloped

**Figure 6:**
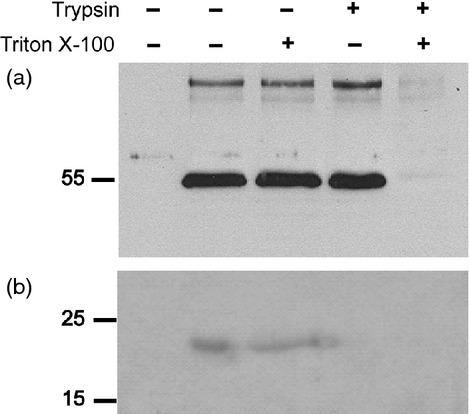
Gag, but not dgp41, is protected from trypsin digestion, suggesting the proteins are organized into enveloped VLPs. Following density centrifugation, the Gag/dgp41-enriched fraction was subjected to proteolysis in the presence or absence of 1% Triton-X-100, subjected to SDS-PAGE and immunoblotting. (a) Detection with anti-p24 antiserum. (b) Detection with 2F5 monoclonal Ab against MPER. The first lane (from left) contains proteins from noninfiltrated wild-type plant extract. The remaining lanes contain VLP-enriched samples from a transgenic Gag-expressing plant, incubated as indicated with or without Triton-X-100 and/or trypsin.

The fact that dgp41 changes its sedimentation pattern upon co-expression with Gag, and especially its shift to a denser fraction cosedimenting with Gag, suggests that these proteins could be colocalized to the same particles, in turn suggesting that the particles should be enveloped. This hypothesis was tested by subjecting Gag/dgp41 preparations, enriched by optiprep gradient centrifugation, to controlled proteolysis in the presence or absence of detergent to disrupt the membrane. Our results (Figure 6) demonstrated that in the absence of the detergent Triton-X-100, plant-derived Gag protein was protected from trypsin digestion. However, when the membrane was compromised by the detergent, Gag was no longer protected, strongly suggesting that the protein was enclosed within membrane vesicles. Importantly, the MPER moiety of gp41 was digested by trypsin whether detergent was present or not, indicating it was exposed at the external face of the membrane.

### HIV-1 VLPs can be directly observed *in situ*

**Figure 7:**
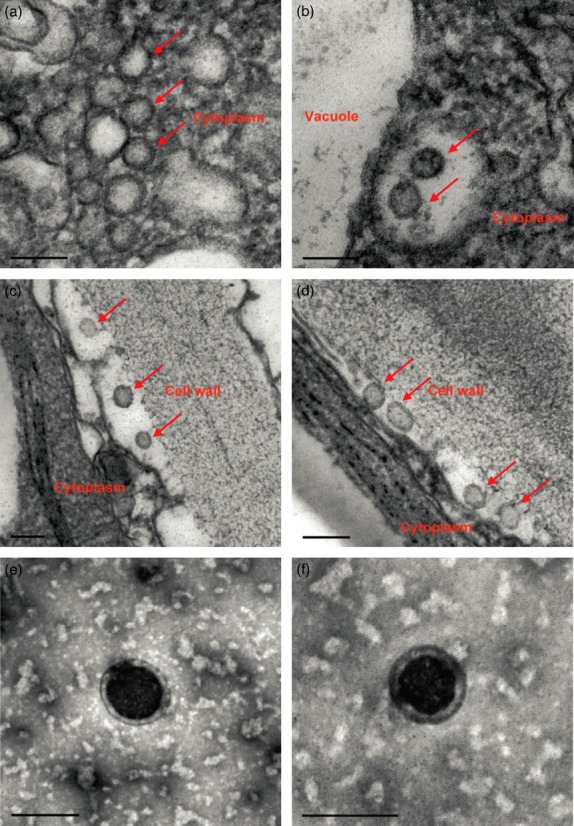
Gag enveloped VLPs observed *in situ* by TEM. (a–d) Representative sections from chemically fixed transgenic gag leaf tissue showing VLP production accumulation (red arrows) in cytoplasm (a), in endosomes near the vacuole (b) and within the apoplastic space (c and d). (e and f) Representative negatively stained VLPs from VLP-enriched extract of transgenic gag leaves. Bar represents 100 nm.

Transmission electron microscopy (TEM) was performed on both the leaf extracts and intact leaf tissue of both stably and transiently transformed Gag-expressing plants. In leaf extracts, enveloped VLPs with diameters of ∼100 nm were visualized (Figure 7e,f). Similar-sized particles could also be observed *in situ* in both transiently and stably expressed leaf tissue (Figure 7a–d). VLPs were found in the apoplastic space between the cell wall and the plasma membrane (Figure 7c,d), but also within an intracellular compartments such the lumen of cytoplasmic membrane vesicles (Figure 7b) and perhaps even in the cytoplasm itself (Figure 7a).

Moreover, in tissues of plants that express the Gag protein, but not in tissues from untransformed plants (data not shown), we observed electron-dense patches on various cellular membranes that we interpreted as congregating Gag protein molecules during the process of VLP budding across the plasma membrane out into the apoplastic space. Similar structures were observed in mammalian (Hammonds *et al.*, 2007), insect (Tagliamonte *et al.*, 2010) and yeast cells (Sakuragi *et al.*, 2002) that express Gag from HIV-1 and other lentiviruses.

Virus-like particles from leaf tissue of plants co-expressing Gag and dgp41 were also observed in both leaf tissue and enriched VLP-containing extracts, and these VLPs were morphologically similar to those found in the Gag-only expressing plants (data not shown).

### Budding of Gag into protoplast medium

**Figure 8:**
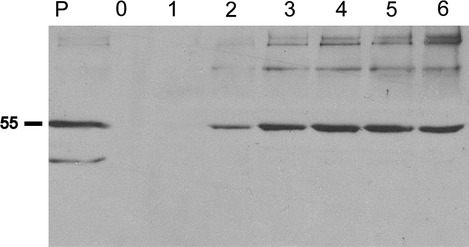
Gag VLPs are released extracellularly as a function of time. Protoplasts were prepared from leaves of Gag-expressing transgenic plants and were then incubated in isotonic protoplast medium for up to 6 h and samples were taken hourly from the medium as indicated. P – Protoplast extract showing total amount of Gag within the cells.

Our observation by TEM of HIV-1 Gag and Gag/dgp41 VLPs budding across cellular membranes, especially across the plasma membrane prompted us to hypothesize that such VLPs could be collected from extracellular medium upon their export out of the cells. However, we expected these relatively large structures to become trapped in the apoplastic space between the plasma membrane and the cell wall. To facilitate testing our hypothesis, we removed the constraint of the cell wall by its enzymatic digestion to generate protopolasts from transgenic Gag plants. These protoplasts were incubated in culture medium, which was sampled every hour up to 6 h after protoplast isolation to check for the presence of Gag protein (Figure 8). Protoplasts remained intact for the duration of the 6 h of incubation, and lysis was apparent only after longer incubation periods (data not shown). We observed time-dependent accumulation of Gag protein in the protoplast medium (separated from the protoplasts by gentle short centrifugation). The initial supernatant (fresh buffer with newly isolated protoplasts) did not contain any Gag protein. The presence of Gag was detected at 2 h and their levels continued to increase as a function of the incubation time.

Importantly, only intact Gag protein was found in the medium, whereas intracellular accumulation of the protein was always accompanied by the accumulation of truncated forms/degradation products. Specifically, a 44-kD band was found in the cells but not in the incubation medium. These results strongly suggest that Gag, not a secretory protein, is released from the cells, probably in the form of VLPs that bud out of the plant plasma membrane into the medium. Taken together with the TEM pictures showing VLPs budding into the apoplastic space *in vivo*, it can be concluded that plant cells can support the formation and budding of HIV-1 Gag VLPs.

## Discussion

Our laboratory has been exploring the immunologically important MPER domain of the transmembrane envelope protein of the HIV-1 virus, gp41 as a plant-derived vaccine antigen through its fusion to the mucosa-targeting CTB protein (Matoba *et al.*, 2004, 2006, 2008, 2009). However, the unique immunological properties of the MPER domain that make it an attractive vaccine target may stem from its proximity to- and its possible interactions with the phospholipid membrane that can be found only in enveloped virions or enveloped VLPs. The work presented here was aimed to explore the hypothesis that plants can accumulate immunogenic HIV-1-enveloped VLPs consisting of the main structural protein of the virus, Gag surrounded by a membrane in which a deconstructed version of gp41 is correctly anchored with its MPER domain exposed. We have brought evidence that plants can indeed express these two recombinant proteins (Figures 1–4 and 8) and that they assemble into enveloped VLPs (Figures 4 and 6) that can bud out of the cell (Figures 6 and 7).

Plant expression of derivatives of p55^Gag^ (including p24, p17 and smaller determinants) was previously attempted with varying degrees of success using both stable (Gonzalez-Rabade *et al.*, 2011; Kim *et al.*, 2004; Lindh *et al.*, 2008; McCabe *et al.*, 2008; Meyers *et al.*, 2008; Obregon *et al.*, 2006; Shchelkunov *et al.*, 2006; Zhang *et al.*, 2002) and transient transformation techniques (Meyers *et al.*, 2008; Regnard, 2010; Perez-Filgueira *et al.*, 2004; Zhang *et al.*, 2002). The more successful cases of those efforts relied on plant-virus vectors (Perez-Filgueira *et al.*, 2004) and expression in transplastomic plants (Gonzalez-Rabade *et al.*, 2011; McCabe *et al.*, 2008).

Even fewer, only two in fact, studies have been published that describe the accumulation of full-length p55^Gag^ in plants (Meyers *et al.*, 2008; Scotti *et al.*, 2009) and both studies reported little or no success when the protein was directed to accumulate in its natural subcellular compartment, the cytoplasm. Better accumulation levels were reported when the protein was expressed in chloroplasts (i.e. transplastomic plants) or targeted to the organelles following transient or stable nuclear transformation (Meyers *et al.*, 2008; Scotti *et al.*, 2009). Both studies reported lack of success to demonstrate full-length p55^Gag^ in its natural subcellular compartment, the cytoplasmic face of the plasma membrane (Meyers *et al.*, 2008; Scotti *et al.*, 2009). Stable transformants, whether harboring a nuclear or plastidic *gag* transgene, showed signs of stress that resulted in ‘[o]verall, Pr55Gag expression [that] was negligible compared with that of p24 and p17/p24’ in the case of transgenic plants (Meyers *et al.*, 2008) or in greatly reduced fitness under greenhouse conditions in the case of transplastomic plants (e.g. chlorosis and reduced productivity, Scotti *et al.*, 2009). In contrast, our approach led to successful accumulation of the full-length Gag protein in the cytoplasm, at more than 20 mg/kg—levels that were >400-fold higher than those reported by Meyers *et al.*, (2008) for p55^Gag^ that was post-translationally targeted to the chloroplast. Moreover, while the levels of accumulation we report here are about 10-fold lower than those reported by Scotti *et al.*, (2009) for transplastomic plants under greenhouse conditions, our plants did not exhibit any signs of stress under normal cultivation routines. The differences between these reports and the current one are difficult to assess in the absence of side-by-side systematic studies or meta-analysis of published research (see Appendix S1).

Stable transgenic lines that express Gag provided us the needed tool for the co-expression of both Gag and dgp41 (MPER, transmembrane and cytoplasmic domains of gp41). This feat was achieved by employing our Gag-expressing plants for the transient expression using one of the most robust systems available to date, the MagnICON system. While the MagnICON system was used with transgenic host plants before (Strasser *et al.*, 2009), to our knowledge, such a combination of transgenic and transient expression has not been reported for proteins that form a complex whose partners are expressed by the two gene expression modalities.

Co-accumulation of the two proteins seemed to stabilize the expression of both Gag and dgp41. This can be easily explained if the two proteins interact within the context of an enveloped VLP as we have demonstrated here for the first time for plants. The ability of plants to support enveloped VLPs is not immediately apparent. Only a handful of enveloped plant viruses were described belonging to just two families—rhabdoviridae and bunyaviridae (Ammar *et al.*, 2009; Kormelink *et al.*, 2011; Kuzmin *et al.*, 2009; Pappu *et al.*, 2009; Walter and Barr, 2011) and not much is known about the molecular biology of the assembly and budding of these viruses. Nonetheless, it seems likely that plants have the machinery needed for enveloped VLP production, which is supported by the fact that the 19 species of the enveloped plant-virus genus *Tospovirus* have one of the widest ranges of hosts (over 800 plant species) of any plant-virus (Adkins *et al.*, 2005; Pappu *et al.*, 2009).

At least some of the genes (and subsequent gene products) that are necessary for the assembly and budding of HIV-1 VLPs from the cell have homologues in plants. For example, in order for VLPs to form and bud from the cell, an N-terminal myristoyl group must be covalently attached to the terminal glycine of the matrix portion of the Gag protein by N-myristoyl-transferase (NMT) during translation, which increases membrane affinity of Gag. Most eukaryotes have two NMT genes (Podell and Gribskov, 2004), which have been sufficient for VLP production in mammalian, insect and yeast cell cultures. Plants express several myristoylated proteins and plant homologues of animal NMTs have been described (Figure S4, Podell and Gribskov, 2004).

In addition to myristoylation, targeting of Gag to the plasma membrane also requires the presence of phosphatidylinositol 4,5-bisphosphate (PI(4,5)P_2_)_,_ a minor phospholipid located at the plasma membrane in mammals (Campbell *et al.*, 2001; Patil *et al.*, 2010; Saad *et al.*, 2007). Although PI(4,5)P_2_ in higher plants is found at lower levels than those found in animals, it seems to be important for vesicular trafficking and cytoskeleton regulation (Meijer and Munnik, 2003). Interestingly, it is not confined to the plasma membranes, perhaps explaining the apparent presence of Gag in other cellular membranes, such as the vacuolar membrane (Figure 7). However, it has been shown that the phosphoinositide becomes concentrated at the plasma membrane in response to certain environmental conditions (e.g. salt stress), developmental cues (at the tips of growing root hairs for example) or biochemical treatments (like phospholipase C inhibition, van Leeuwen *et al.*, 2007). When it is localized to the plasma membrane, it is mostly concentrated at membrane rafts (Furt *et al.*, 2010) as is the case in mammalian cells (Holm *et al.*, 2003), meeting another condition for targeting Gag to the plasma membrane in plant cells and suggesting a way to increase the efficiency of the process.

One of the most evocative finding in the work presented here is the observation that VLPs cannot not only form in the plant cell, but also bud out. The endosomal sorting complex required for transport (ESCRT) plays an important role in the budding of HIV-1 from mammalian cells (Jager *et al.*, 2007; Pincetic and Leis, 2009). Two epitopes, PTAP and YPLTSL, on the C-terminus of Gag’s p6 domain are involved in the ESCRT pathway (Strack *et al.*, 2003). PTAP (which is dominant) binds the cellular ESCRT protein, Tsg101 (Stuchell *et al.*, 2004), and YPLTSL binds ALIX/AIP1 (Strack *et al.*, 2003). Plants contain orthologues for all major ESCRT complex subunits, including Tsg101 (Figure S5) and ALIX/AIP1 (Figure S6, Otegui and Spitzer, 2008; Spitzer *et al.*, 2009), and it can be speculated that budding of HIV-1 VLPs from plant cells’ plasma membrane (Figures 6 and 7) is aided by the endogenous plant ESCRT pathways. Budding of enveloped VLPs from plant cells was also recently demonstrated for influenza virus haemagglutinin-based VLPs (D’Aoust *et al.*, 2010).

The results presented here, considered *in toto*, suggest that the availability of plant-based HIV-1 Gag/dgp41 VLPs should enable their evaluation as an effective component of a vaccine against HIV-1 (Kessans, Matoba and Mor, manuscript in preparation). As plant-made VLPs are yet to obtain regulatory approval, the extent of their purity remains to be determined. To use VLPs as HIV vaccines, future studies need to develop a more economical and scalable downstream processing procedure using simple filtration and chromatographical techniques to replace the ultracentrifugation-based procedure employed in this proof-of-concept work.

## Experimental procedures

### Plant optimization and *de novo* construction of the gag and dgp41 genes

The gag gene (from subtype C R5 HIV-1 isolate, 1084i, GenBank Accession no. AY805330) containing the coding sequences for the entire p55^Gag^ protein (Figure 1) was optimized for expression in *N. benthamiana* using methods previously described (Geyer *et al.*, 2010). Changes made to the sequence in order to obtain the newly designed gene (Figure S1, GenBank Accession number JX534517) are listed in Table S1. A total of 29 forward and 29 reverse partially overlapping oligonucleotides were assembled via assembly PCR using the Expand High Fidelity PCR kit (Roche). The primers at the 5′ and 3′ end of the gene were then used to amplify the entire gene (Figure S1).

The deconstructed gp41 gene was designed as a chimera consisting of the gp41 MPER derived from the B-clade MN isolate (GenBank Accession number AF075722) and the transmembrane domain and cytoplasmic tail region of the C 1084i isolate (GenBank Accession number AY805330). The gene (GenBank Accession number JX534518) was plant optimized as described earlier and was synthesized by Integrated DNA Technologies.

The assembled synthetic genes were cloned into the PCR-cloning vector, pTOPO-TA (Invitrogen) to create pTM445 and pTM601 for *gag* and *dgp41*, respectively. The restriction sites *NcoI* and *SacI* were added to the 5′ and 3′ ends of the genes (Figure 1), respectively.

### Gag stable expression

The *gag* construct from pTM445 was cloned into the binary vector, pGPTV-Kan (Becker *et al.*, 1992) as previously described (Mor *et al.*, 2001) to create pTM535. Stable transgenic *N. benthamiana* expressing Gag were established by cocultivation of *A. tumefaciens* LBA4404 harbouring pTM535 with sterile leaf explants (>300) selection for kanamycin resistance and regeneration as previously described (Geyer *et al.*, 2007). Gag expression in regenerated plants was confirmed by SDS-PAGE and immunoblotting and regenerated Gag-expressing plants were transferred to soil for seed generation.

### Transient expression of dgp41

A deconstructed TMV Vector system (MagnICON, used by kind permission of ICON Genetics under material transfer agreement) was used for transient expression of dgp41 in *N. benthamiana* (Marillonnet *et al.*, 2005). To this end, the NcoI-SacI restriction fragments from pTM601 were cloned into the 3′ module of the ICON system (pICH11599) as previously described (Huang *et al.*, 2006) to yield pTM602, which was introduced into competent *A. tumefaciens* LBA4404 (Figure 1). Targeting the ER (and consequently the plasma membrane) was achieved with a 5′-provirus module that contained the barley alpha-amylase signal peptide (pICH20999, Kalthoff *et al.*, 2010). For transient expression, *A. tumefaciens* LBA4404 cell lines harbouring the modules of the viral vector pTM602 were grown to logarithmic phase, resuspended in infiltration buffer (10 mm MES, 10 mm MgSO_4_, pH 5.5) at a final optical density at 600 nm of 0.01 and inoculated into 6-week-old *N. benthamiana* plants by vacuum infiltration. Plants were inverted into a vacuum container containing 2 L of *A. tumefaciens* and a vacuum of -600 mmHg was applied to the container for 2 min. The vacuum was quickly released, allowing the *A. tumefaciens* to infiltrate the leaf. Infiltrated plants were kept in a controlled environmental growth chamber at 25 °C until harvest, and the presence of dgp41 in leaf extracts was confirmed by SDS-PAGE and immunoblotting.

### SDS-PAGE and immunoblotting

For immunoblotting, leaf extract was homogenized with plant extraction buffer in a ratio of 1 mg leaf tissue: 3 μL extraction buffer [25 mm Na_2_HPO_4_/NaH_2_PO_4_, 100 mm NaCl, 1 mm ethylenediaminetetraacetic acid (EDTA), pH 7.8] and a ceramic bead in a Fast Prep-24 (MP Biomedicals, Solon, OH) machine for 40 s. Extract was clarified at 12 000 ***g*** for 10 min and the supernatant was collected. Extract (200 μL) was added to 5x SDS loading buffer (40 μL, 30% glycerol, 35 mm SDS, 60 mm dithiothreitol (DTT), 18 mm bromophenol blue, 350 mm Tris-HCl, pH 8.0) and incubated for five min at 100 °C to denature the proteins. Polyacrylamide gels (12%) were prepared, and each sample (20 μL) was loaded into its respective well. Gels were run at 40 mV until proteins were separated. Proteins were then transferred to nitrocellulose membrane and blocked with phosphate-buffered saline (PBS, 10 mm Na_2_HPO_4_, 1.8 mm KH_2_PO_4_, 2.6 mm KCl, 135 mm NaCl, pH 7.4) supplemented with tween-20 (0.05%, v/v, PBST) and 5% (w/v) dry milk (PBST-M) for 1 h at 25 °C. Membranes were then incubated for 1 h in PBST-M containing either a polyclonal anti-p24 rabbit serum (for detection of Gag protein, see Appendix S1) or the human monoclonal 2F5 antibody, a kind gift from Morgane Bomsel (for the detection of dgp41). Membranes were washed 3 × 30 min with PBST and then incubated with PBST-M in the presence of a secondary antibody conjugated to HRP (anti-rabbit IgG for p24 and anti-human IgG for 2F5) for 1 h at 25 °C. Membranes were then washed 3 × 30 min with PBST, and Abs were detected using enhanced chemiluminescence (ECL) with ECL reagent (Santa Cruz) and exposed to film.

The quantification of proteins was performed with quantitative immunoblotting. Pure samples of either the fusion protein p24-CTA2 (the p24 peptide genetically-fused to the CTA2 domain of cholera toxin, see Appendix S1) or CTB-MPR (Matoba *et al.*, 2008) were used as standards that were quantified spectrophotometrically (*A*_*260*_) using the following extinction coefficients: 1.22 mm/cm (p24-CTA2) and 2.22 mm/cm (CTB-MPR). Samples of the standard proteins (at 10 μL) containing 50, 40, 30, 20, or 10 ng of p24 (80% mass of p24-CTA2) or the MPER peptide (33% mass of CTB-MPR) alongside appropriately diluted samples of proteins to be quantified were resolved by SDS-PAGE followed by immunoblotting as described previously. Densitometry of scanned blot images was performed using the ImageJ software. Concentrations of experimental samples were calculated from equations derived by linear regression of the standard curves.

### Optiprep density sedimentation

Further characterization and purification of VLPs was performed using Optiprep (60% iodixanol in water, Sigma Aldrich, St. Louis, MO) density gradient sedimentation. Ultra Clear tubes (14 × 89 mm, Beckman-Coulter, Miami, FL) were layered from the bottom with 1.5 mL each of 60%, 50%, 40%, 30%, 20%, and 10% iodixanol. Clarified, water-soluble leaf extract (3 mL) was layered on the top of the Optiprep gradient, and the tube was spun at 209 490 ***g*** in a SW41Ti rotor (Beckman-Coulter, 209,490 × ***g***) for 5 h at 4 °C. Fractions (1 mL) were collected from the top of the gradients, analysed as described earlier, and VLP-enriched fractions were used in further experimentation.

### Trypsin digestion assay

Enriched fractions containing VLPs were digested with trypsin in the presence or absence of 1% Triton-X-100 (v/v) as per Sakuragi *et al.* (2002). VLP-enriched fractions, containing 200 μg/mL p24 obtained from Optiprep gradient centrifugation, were aliquoted (1 mL) to each of four 1.5-mL centrifuge tubes. One tube remained a negative control, while 1% Triton-X-100 or trypsin (final concentration 1 μg/mL) were added separately to one tube each, and both 1% Triton-X-100 or trypsin (final concentration 1 μg/mL) were added to the final tube. Extract was incubated at 26 °C for 30 min and was then analysed for Gag and dgp41 protein by immunoblotting.

### Transmission electron microscopy

For visualization of whole VLPs, clarified extract from transgenic Gag plants or Gag/dgp41 plants (6 days postinfiltration for dgp41) were incubated for 2 min on 200 mesh formvar-coated grids and stained by incubating grids containing sample with 2% (w/v) uranyl acetate for 2 min. For visualization of VLPs *in situ*, leaf tissue (cut into 1 mm^2^ sections) from Gag- or Gag/dgp41-expressing plants was chemically fixed in primary fixation buffer (2% (v/v) gluteraldehyde, 0.1 m Na_2_HPO_4_/NaH_2_PO_4_, pH 6.8) for 2 h at 25 °C. Following primary fixation, tissue was washed with phosphate buffer (pH 6.8) and incubated in secondary fixation buffer [2% (w/v) osmium tetroxide, 0.1 m Na_2_HPO_4_/NaH_2_PO_4_, pH 6.8] for 2 h at 25 °C. Following fixation, samples were washed three times with phosphate buffer (pH 6.8) to remove osmium tetroxide and then incubated with 0.5% (w/v) uranyl acetate for 2 h at 25 °C to stain samples. Stained samples were completely dehydrated with ethanol (10 min incubations with five increasing concentrations of ethanol for a total of 60 min dehydration time) and then infiltrated with a 1: 3 ratio of Spurr’s resin/acetone. After a 4-h incubation in 1 : 3 Spurr’s resin/acetone, samples were moved to a 1 : 1 Spurr’s resin/acetone mixture for another 4-h incubation at 25 °C. Additional 4 h incubations with 3 : 1 and 100% Spurr’s resin were performed to completely embed the samples in resin. Fresh resin was then added to samples, and the mixture was placed into moulds and heated in a 60 °C oven for 24 h. Sections (70 nm) from these samples were cut on diamond knives, positioned on formvar-coated grids and stained with 2% uranyl acetate and 2% lead acetate as above. Specimen grids were viewed on a Philips CM12S transmission electron microscope.

### Protoplast experiments

Leaves (10 g) of transgenic Gag-expressing plants were surfaced sterilized with 70% ethanol, rinsed in water, cut into ∼1 cm^2^ sections and immersed in protoplast isolation buffer (20 mL, 0.625 m sucrose, 25 mm MES, pH 5.7) containing cellulase (2.5 mg/mL) and pectinase (4 mg/mL) for 1 h at 25 °C. The protoplast isolation buffer was then carefully siphoned off and replaced with fresh isolation buffer and gently shaken (50 rpm) for 5 min. The solution containing released protoplasts was then centrifuged at 200 ***g*** for 5 min to pellet any remaining cell debris and the supernatant containing live protoplasts was carefully transferred to a sterile Petri dish and incubated for 6 h at 25 °C with gentle shaking (50 rpm), with supernatant samples taken at the indicated time points for further analyses.

## Supporting information

**Figure S1** Sequence alignment of native and plant-optimized *gag* genes and their expected translation product. **Figure S2** Sequence alignment of native and plant-optimized *dgp41* genes and their expected translation product. **Figure S3** Codon usage of plant-optimized *gag* and dgp41 is comparable with that of a highly expressed plant gene. **Figure S4** Protein sequences homologues to NMT-1 found in humans, *Arabidopsis* thaliana, and rice were aligned using the T-Coffee programme, and the degree of similarity is shown as a heat diagram superimposed on the sequences. **Figure S5** Protein sequences related to Tsg101 found in humans, Arabidopsis, and rice were aligned using the T-Coffee programme, and the degree of similarity is shown as a heat diagram superimposed on the sequences. **Figure S6** Protein sequences related to Alix found in humans, Arabidopsis and rice were aligned using the T-Coffee programme, and the degree of similarity is shown as a heat diagram superimposed on the sequences. **Appendix S1** Biological and biochemical characterization of HIV-1 Gag/dgp41 virus-like particles expressed in *Nicotiana benthamiana*.
