# Supplementary material for: Biological and biochemical characterization of HIV-1 Gag/dgp41 virus-like particles expressed in Nicotiana benthamiana
Source: Plant Biotechnol J. 2013 Mar 19;11(6):681–90. doi: 10.1111/pbi.12058 (PMC3688661; doi:10.1111/pbi.12058)

# Biological and biochemical characterization of HIV-1 Gag/dgp41 virus-like particles expressed in Nicotiana benthamiana.

Sarah A. Kessans^1^, Mark D. Linhart^1^, Nobuyuki Matoba^1,2,3,*^ Tsafrir Mor^1,*^

^1^School of Life Sciences and The Biodesign Institute, P.O. Box 874501, Arizona State University, Tempe, AZ, 85287-4501

^2^Owensboro Cancer Research Program, Owensboro, KY 42303, and ^3^James Graham Brown Cancer Center and Department of Pharmacology & Toxicology, University of Louisville School of Medicine, Louisville, KY 40202

^*^Co-Corresponding Authors:

Tsafrir S. Mor
School of Life Sciences and The Biodesign Institute
P.O. Box 874501
Arizona State University
Tempe, AZ 85287-4501
Tel: 480-727-7405; Fax: 480-965-6899

Nobuyuki Matoba
James Graham Brown Cancer Center and University of Louisville School of Medicine
1020 Breckenridge Street, Suite 201
Owensboro, KY 42303
Tel:  270-691-5955; Fax:  270-685-5684

Email: [tsafrir.mor@asu.edu](mailto:tsafrir.mor@asu.edu)
 [Sarah.Kessans@asu.edu](mailto:Sarah.Kessans@asu.edu)
 [mlinhart@asu.edu](mailto:mlinhart@asu.edu)
 [n.matoba@louisville.edu](mailto:n.matoba@louisville.edu)

Running title: Plant-derived HIV-1 Gag/dgp41 virus-like particles

Key words: HIV-1; enveloped virus-like particles; transgenic plants; transient expression; Gag; gp41

Accession numbers: JX534517, JX534518

Word Count: Summary: 189
 Entire Paper (without references and supplemental): 6998

## SUPPLEMENTAL MATERIALS: MATERIALS AND METHODS

### Generation of Anti-p24 Antibodies

In order to develop anti-p24 antibodies, a fusion construct consisting of the plant-optimized p24 gene and the cholera toxin A2 subunit (CTA2) was developed. The gene for p24 was PCR amplified from the plant-optimized *gag* gene with primers oTM374 and oTM375 , which added the FacsI and FseI restriction sites to the 5’ and 3’ ends of the gene, respectively. The amplified gene product was cloned into pTM319, a pET-22b(+) *E. coli* expression vector containing the gene for CTA2, to create pTM 460. The p24 gene was cloned in-frame upstream of the CTA2 gene. The p24-CTA2 fusion construct was expressed in *E. coli* BL21 (DE3) cells and purified as previously described (Matoba et al., 2008). Briefly, *E. coli* cultures containing pTM 460 were grown from single colonies overnight at 37°C. Logarithmic phase cultures were induced with 100 μM isopropyl β- D -1-thiogalactopyranoside (IPTG) and allowed to grow for 2 h before centrifugation of the cells. Cell pellets were resuspended in PBS (20 ml) and lysed by microfluidization. Lysed cells were pelleted by centrifugation, and the insoluble pellet was solublized in 8M urea buffer (50 mM Tris, 500 mM NaCl, 8 M urea, pH 8.0), rocked at 4°C for 60 min, centrifuged to remove the remains of the insoluble cell fractions, and dialyzed in PBS with a 10 kD-cutoff membrane for 24 h to remove urea. The soluble supernatant was then added to a 3 ml bed volume Talon gravity flow column and purified by metal affinity chromatography. The final column eluate (6 ml) was dialyzed in PBS with a 10 kD-cutoff membrane for 24 h to remove imidizole. Protein remained souble throughout the dialysis. Purity and quantification of the pure p24-CTA2 was determined by Coomassie stained gels, immunoblots, and by determining the absorbance at 280 nm using (ɛ = 1.22 mM^-1^ cm^-1^). Two New Zealand white rabbits were inoculated with 4 subcutaneous injections of 200 µg p24-CTA2 emulsified with Freund’s complete adjuvant (100 µl at 4 sites per rabbit, 0.5 µg/µl p24-CTA2 in sterile PBS). Rabbits were boosted with 4 subcutaneous injections of 200 µg p24-CTA2 emulsified with Freund’s incomplete adjuvant (100 µl at 4 sites per rabbit, 0.5 µg/µl p24-CTA2 in sterile PBS) two weeks after the initial inoculation. The level of antibody response was determined by p24 ELISA (explained in detail in Chapter 4), and the rabbits were exsanguinated upon peak response observed (two weeks following boost). More than 120 ml of blood was collected from each rabbit, and the serum (>90 ml/rabbit) was separated from the red blood cells by centrifugation at 4,000 g for 10 min. Serum was allocated into 1 ml fractions and stored at -80°C until use.

## SUPPLEMENTAL MATERIALS: DISCUSSION

### Comparative analysis of expression levels

What can explain our relative success, and in particular what may be the differences between the two previous studies (Meyers et al., 2008; Scotti et al., 2009) involving cytoplasmic accumulation of full-length Gag expressed from a stable nuclear transgene and the results presented here? Despite the different agrobacterial strains, binary vectors, or control elements that were used by the different groups, these were all standard tools, employed routinely and often with good success in conjunction with other recombinant genes and we can dismiss their involvement in limiting the expression levels as unlikely. While perseverance during the screening process may be responsible for our ability to identify 2 Gag-expressing plants among many independent transformation events (neither of the other two reports specify how many explants were transformed and screened), we do not think that this is a compelling explanation. In fact, meager accumulation levels were reported by the two other groups also for their transient agroinfiltration studies, unlike our experience with our constructs.

We should next consider the actual transgene sequences. Scotti et al. utilized a Clade B *gag* gene as their expression target, unlike Meyer et al. and us who used a Clade C *gag* gene. The native cDNAs of these genes (difficult to assess in the case of Scotti et al who do not provide the accession number for the gene) are rather AT rich (close to 60%), which may explain how favorable was the transplastomic expression of the gene. While Scotti et al. opted to use only the native cDNAs (with no codon optimization) for their expression, Meyers et al. reported making use of “plant codon optimisation”. In the absence of an agreed definition of what “codon optimization” entails, the term is rather nonchalantly used in the literature. It would have been beneficial to compare plant-codon optimization strategies between this study and that of Meyer et al., but neither the details of their optimization strategy nor the sequence of the “codon-optimized” gene were published. For this project we have followed our previously published strategy, by adopting the codon usage of the transgene to that common among highly expressed plant genes (Geyer et al., 2007; Geyer et al., 2010). Our algorithm further seeks to remove mRNA destabilizing sequences, premature polyadenylation sequences, cryptic splicing signals and potential methylation sites and typically conforms the GC content to that typical of dicotyledonous plants (50%). In the absence of side-by-side systematic studies or meta analysis of published research, it will be difficult to dispel the commonly held view that heterologous gene expression in plants is more of an art than science that “need[s] to be assessed on a case-by-case basis” (Pelosi et al., 2012). We feel that such studies are essential if we don’t want the “empirical process” (Rybicki, 2010) of honing our plant-expression tools to be reduced to an endless trial-and-error process.

## SUPPLEMENTAL MATERIALS: TABLES

### Table S1

#### Table S1. Molecular features of native and synthetic Gag and dgp41 genes

|  |  | *Gag****^a^*** | *Optimized Gag****^b^*** | *dgp41****^C^*** | *Optimized dgp41****^D^*** |
| --- | --- | --- | --- | --- | --- |
|  |  |  |  |  |  |
| Codon usage |  |  |  |  |  |
|  | Total | 493 | 493 | 216 | 216 |
|  | Unfavorable | 181 | 23 | 81 | 1 |
|  | % | 37 | 5 | 38 | <1 |
|  | Clusters of 2-3  4≤ | 15  5 | 1  0 | 16  4 | 0  0 |
|  | CAI | 0.52 | 0.83 | 0.55 | 0.91 |
|  |  |  |  |  |  |
| RNA destabilizing sequences | |  |  |  |  |
|  | AUUUA* | 1 | 0 | 0 | 0 |
|  | AUAGAU* | 1 | 0 | 0 | 0 |
|  | UUUUUU* | 0 | 0 | 0 | 0 |
|  | 1-10 seqs of Narsai et al. | 2 | 0 | 2 | 0 |
|  |  |  |  |  |  |
| Polyadenylation signals | |  |  |  |  |
|  | 1-10 seqs of Loke et al. | 14 | 0 | 7 | 0 |
|  | 1-100 seqs of Loke et al. | 103 | 15 | 48 | 5 |
|  |  |  |  |  |  |
| Donor splice sites | |  |  |  |  |
|  | Highly likely | 0 | 0 | 0 | 0 |
|  | Potential | 0 | 0 | 0 | 0 |
|  |  |  |  |  |  |
| Acceptor splice sites | |  |  |  |  |
|  | Potential | 0 | 0 | 0 | 0 |
|  |  |  |  |  |  |
| Branch point splice sites | |  |  |  |  |
|  | Potential | 0 | 0 | 0 | 0 |
|  |  |  |  |  |  |
| Potential DNA methylation sites | |  |  |  |  |
|  | CG/CNG | 85 | 36 | 38 | 14 |
|  |  |  |  |  |  |
|  |  |  |  |  |  |
| % GC |  | 44% | 49% | 44% | 45% |
|  |  |  |  |  |  |
|  |  |  |  |  |  |
| **^a^**Accession number: AY805330 **^b^**Accession number: JX534517 **^c^**Accession numbers: MPER - AF075722; transmembrane domain + cytoplasmic tail region - AY805330.  **^b^**Accession number: JX534518 | | | | | |

## SUPPLEMENTAL MATERIALS: FIGURE LEGENDS AND FIGURES

**Figure S1.** Sequence alignment of native and plant-optimized *gag* genes and their expected translation product. Both genes encode for an identical protein (top line), but the nucleotide sequence of the native *gag* was adapted to allow higher levels of expression in designing the plant-optimized *gag* gene (bottom line). Various molecular features that were targeted in optimizing the gene are labeled as shown on the figure.

**Figure S2.** Sequence alignment of native and plant-optimized *dgp41* genes and their expected translation product. Both genes encode for an identical protein (top line), but the nucleotide sequence of the native *dgp41* was adapted to allow higher levels of expression in designing the plant-optimized *dgp41* gene (bottom line). Various molecular features that were targeted in optimizing the gene are labeled as shown on the figure.

**Figure S3.** Codon usage of plant-optimized *gag* and dgp41 is comparable to that of a highly expressed plant gene. Plotted values of the relative adaptiveness, w, of each codon represent a moving average (geometric mean, window size 51) centering on each codon for the coding regions of rbcS1B (encoding the small subunit of the enzyme RuBisCO) and both native and plant-optimized gag (A) and dgp41 (B). The codon adaptation index (CAI, relative adaptiveness averaged over the length of a sequence) for each gene is shown as a broken line.

**Figure S4.** Protein sequences homologs to NMT-1 found in humans, *Arabidopsis* thaliana, and rice were aligned using the T-Coffee program, and the degree of similarity is shown as a heat diagram superimposed on the sequences.

**Figure S5.** Protein sequences related to Tsg101 found in humans, Arabidopsis, and rice were aligned using the T-Coffee program, and the degree of similarity is shown as a heat diagram superimposed on the sequences.

**Figure S6.** Protein sequences related to Alix found in humans, Arabidopsis, and rice were aligned using the T-Coffee program, and the degree of similarity is shown as a heat diagram superimposed on the sequences.

### Figure S1.

*
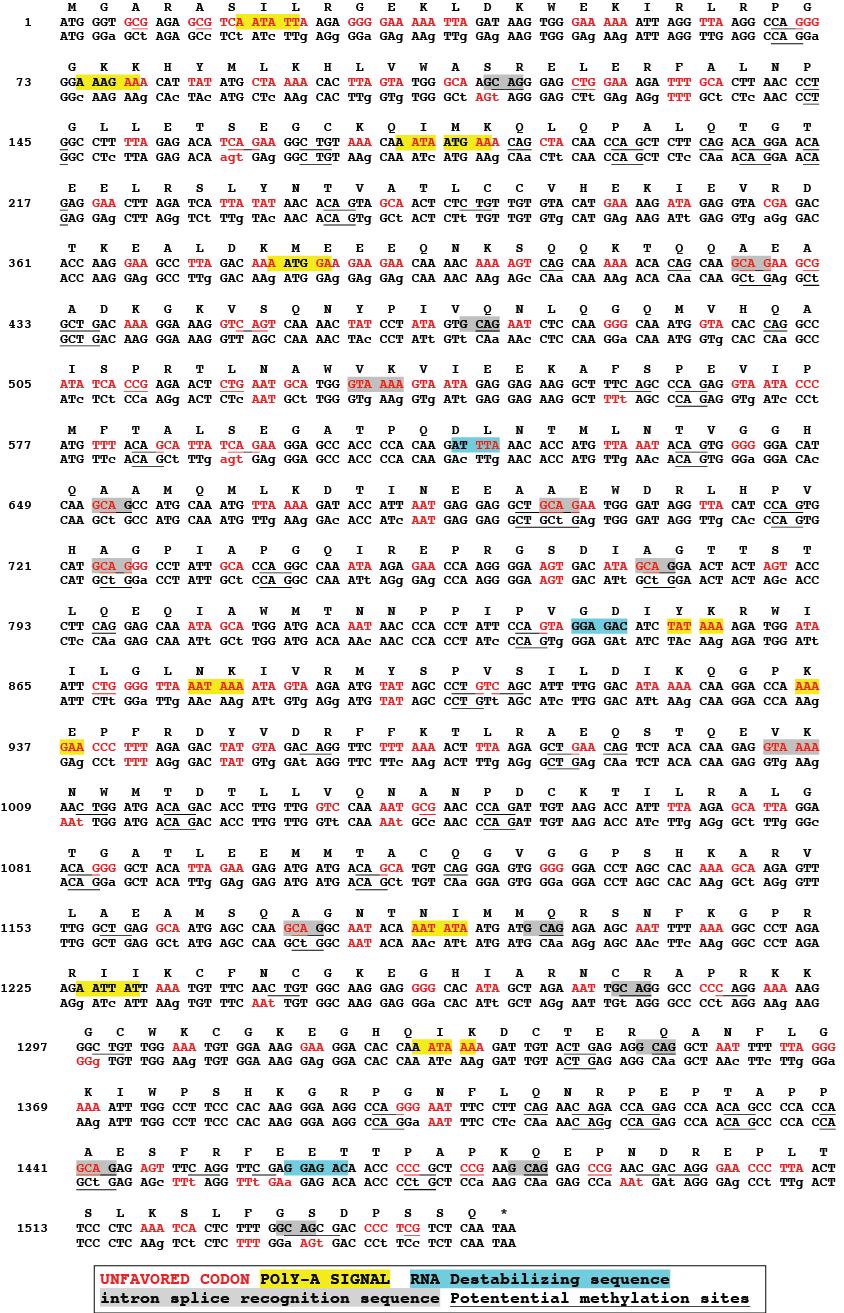
*

*Figure S2.*


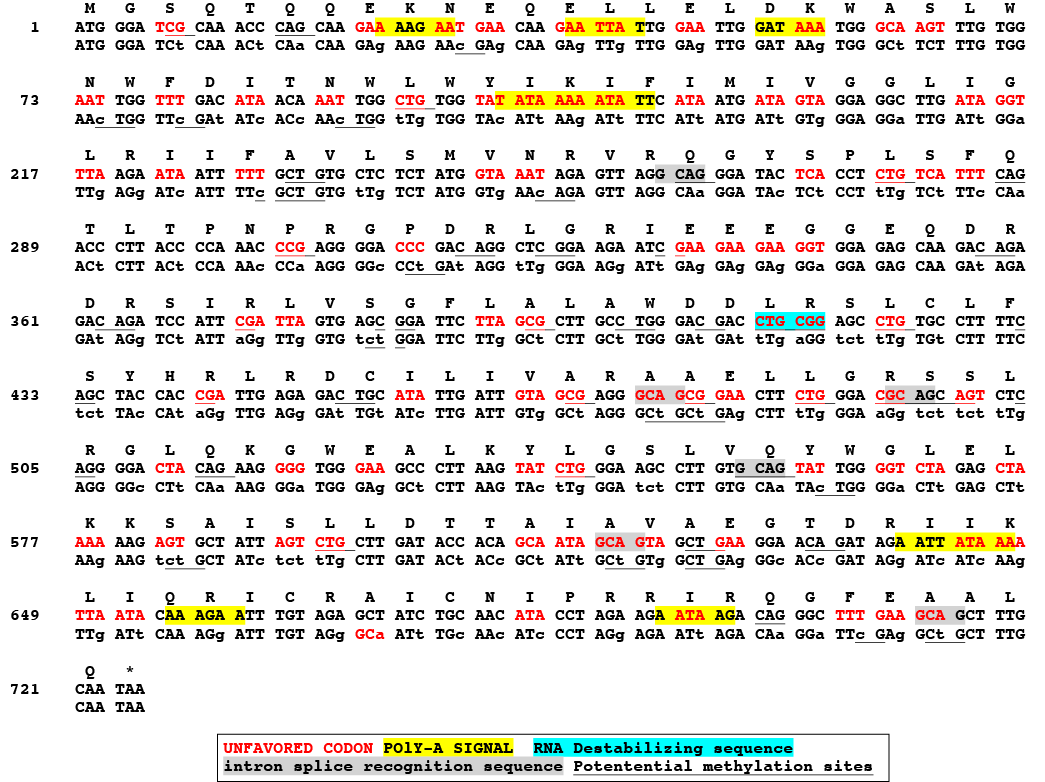


*Figure S3.*


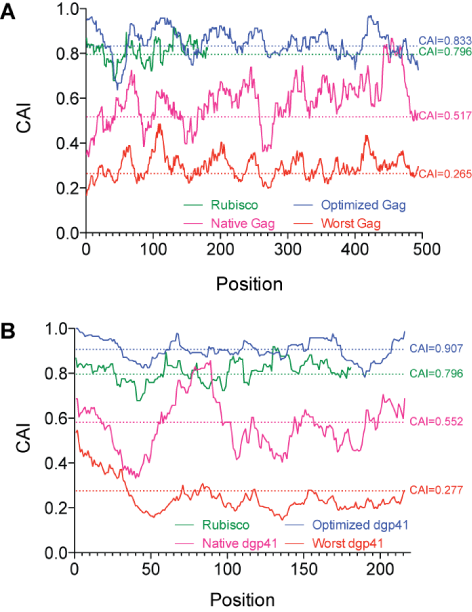


*Figure S4.*


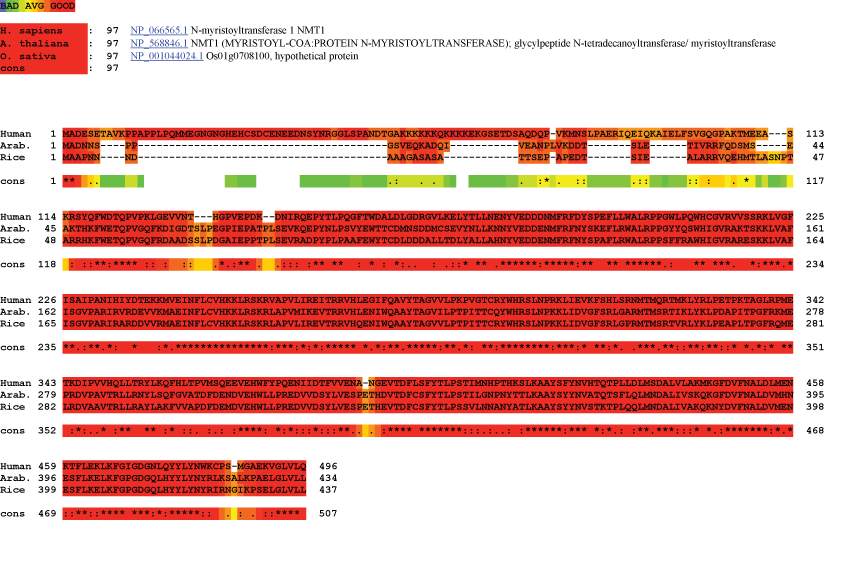


*Figure S5*


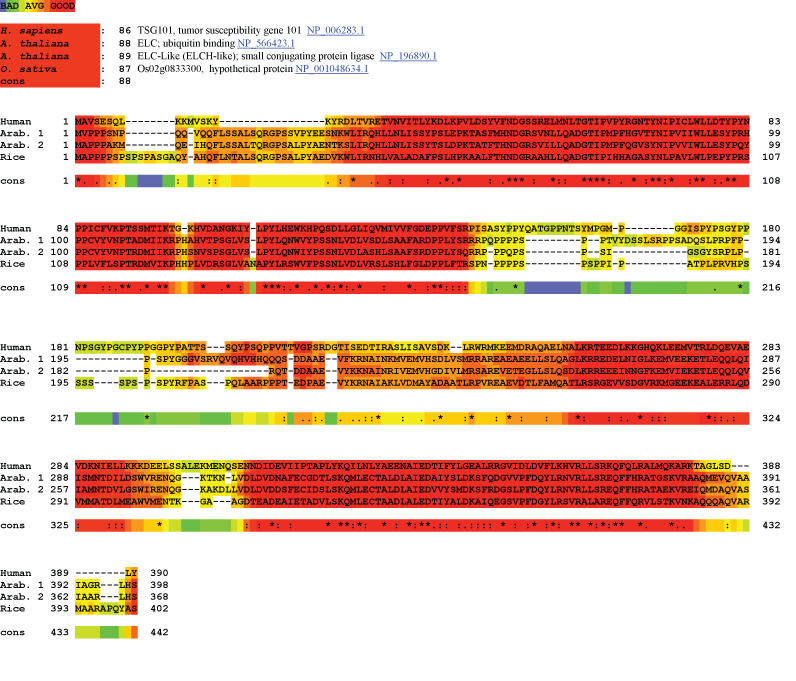


*Figure S6.*


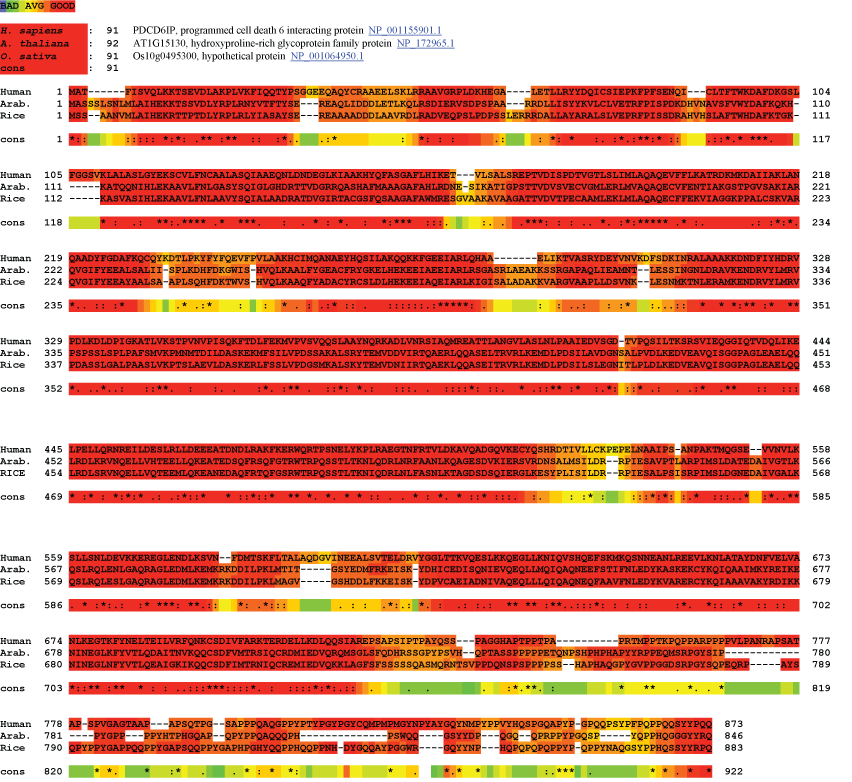

Supplement: Supplementary file 1 — Figure S1 Sequence alignment of native and plant-optimized gag genes and their expected translation product. Figure S2 Sequence alignment of native and plant-optimized dgp41 genes and their expected translation product. Figure S3 Codon usage of plant-optimized gag and dgp41 is comparable with that of a highly expressed plant gene. Figure S4 Protein sequences homologues to NMT-1 found in humans, Arabidopsis thaliana, and rice were aligned using the T-Coffee programme, and the degree of similarity is shown as a heat diagram superimposed on the sequences. Figure S5 Protein sequences related to Tsg101 found in humans, Arabidopsis, and rice were aligned using the T-Coffee programme, and the degree of similarity is shown as a heat diagram superimposed on the sequences. Figure S6 Protein sequences related to Alix found in humans, Arabidopsis and rice were aligned using the T-Coffee programme, and the degree of similarity is shown as a heat diagram superimposed on the sequences. Appendix S1 Biological and biochemical characterization of HIV-1 Gag/dgp41 virus-like particles expressed in Nicotiana benthamiana. [file pbi0011-0681-sd1.docx]
